# Simultaneous Determination of Lamotrigine, Oxcarbazepine, Lacosamide, and Topiramate in Rat Plasma by Ultra-Performance Liquid Chromatography-Tandem Mass Spectrometry

**DOI:** 10.1155/2022/1838645

**Published:** 2022-03-14

**Authors:** Erjie Qiu, Lu Yu, Qishun Liang, Congcong Wen

**Affiliations:** ^1^Department of Clinical Pharmacy, Ningbo YinZhou No. 2 Hospital, Ningbo, China; ^2^Laboratory Animal Centre, Wenzhou Medical University, Wenzhou, China

## Abstract

This study established an ultra-performance liquid chromatography-tandem mass spectrometry (UPLC-MS/MS) method to study the pharmacokinetics of four antiepileptic drugs, lamotrigine, oxcarbazepine, lacosamide, and topiramate, in rats after oral administration. The gradient elution was performed on a UPLC HSS T3 (2.1 mm × 100 mm, 1.8 *μ*m) column with acetonitrile-0.1% formic acid as the mobile phase at a flow rate of 0.4 mL/min. Protein precipitation by acetonitrile was adopted for plasma sample pretreatment. Electrospray- (ESI-) positive/negative ion switching and multiple reaction monitoring (MRM) modes were adopted for ion quantitative determination of antiepileptic drugs. UPLC-MS/MS detection and Drug and Statistics (DAS) software fitting were performed to blood samples collected from rats after oral administration of lamotrigine, oxcarbazepine, lacosamide, and topiramate (5 mg/kg). All drugs examined showed linearity within 5–5000 ng/ml (*R*^2^ > 0.9987), the intraday accuracy was within 92%–108%, and the interday accuracy was within 93%–109%. The relative standard deviations (RSD) of intraday and interday were less than 15%. The matrix effect was within 91%–105%, and the recovery was better than 88%. The established UPLC-MS/MS method was successfully applied to the pharmacokinetic study of lamotrigine, oxcarbazepine, lacosamide, and topiramate in rats.

## 1. Introduction

Epilepsy is a common nervous system disease [1–3]. Epilepsy treatment usually relies on antiepileptic drugs that belong to the symptom control drugs [4,5]. Most epilepsy patients require long-term medication; however, patients show individual differences in drug response [6–10]. To simultaneously improve the effectiveness and safety of clinical drugs and provide a reliable scientific basis for diagnosing and treating drug overdose poisoning, a rapid and accurate method is necessary to determine drug concentration in plasma.

The methods for the determination of antiepileptic drugs mainly include high-performance liquid chromatography (HPLC) [11–14], immunoassay [15, 16], and gas chromatography-mass spectrometer (GC-MS) [17, 18]. Liquid chromatography with tandem mass spectrometry (LC-MS/MS) can obtain strong adduct ion peaks of the compounds under first-order mass spectrometry [19–21]. Although methods for measuring individual antiepileptic drug concentration have been widely reported, some patients with severe conditions require multiple drugs simultaneously. It is urgent to establish a rapid and quantitative screening method for several antiepileptic drugs to meet the clinical needs.

In this study, an ultra-performance liquid chromatography-tandem mass spectrometry (UPLC-MS/MS) method was established to simultaneously determine plasma concentrations of four antiepileptic drugs (lamotrigine, oxcarbazepine, lacosamide, and topiramate) in rats, using midazolam as an internal standard. The proposed method could be helpful for blood concentration monitoring, individual administration plan formulation, drug abuse monitoring, and pharmacokinetic study of these drugs.

## 2. Experimental

### 2.1. Chemicals and Animals

**Figure 1:**
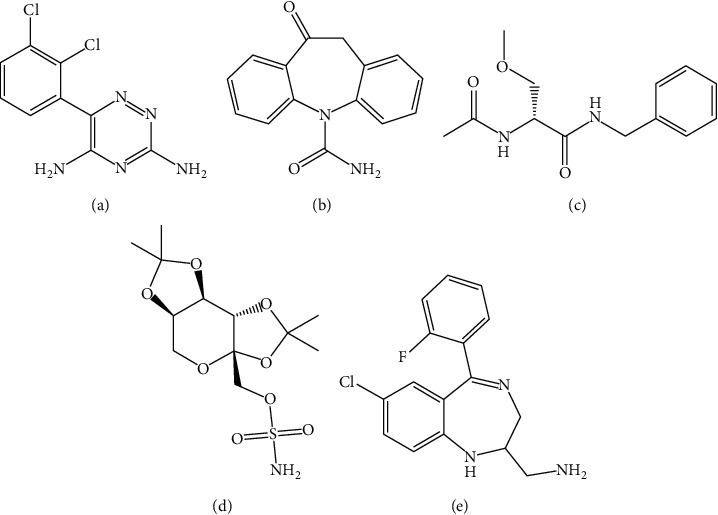
Chemical structure of lamotrigine (a), oxcarbazepine (b), lacosamide (c), topiramate (d), and internal standard (e).

Lamotrigine, oxcarbazepine, lacosamide, topiramate, and midazolam (purity >98%) (Figure 1) were purchased from Beijing Century Aoke Biotechnology Co., Ltd. (Beijing, China). HPLC grade acetonitrile and formic acid were purchased from Merck (Darmstadt, Germany). Ultrapure water was prepared by the Milli-Q water system (Bedford, MA, USA). Sprague-Dawley (SD) rats (male, bodyweight 200–220 g) were obtained from the Animal Experimental Center of Wenzhou Medical University (Wenzhou, China).

### 2.2. Instruments and Conditions

The Acquity H-Class UPLC coupled to the XEVO TQS-Micro triple quadrupole mass spectrometer (Waters Corp., Milford, MA, USA) was used for UPLC-MS/MS. The gradient elution was performed on a UPLC HSS T3 (2.1 mm × 100 mm, 1.8 *μ*m) column (Waters Corp., Milford, MA, USA) with acetonitrile-0.1% formic acid as a mobile phase at a 0.4 mL/min flow rate. The gradient elution conditions were as follows: 0–0.2 min, acetonitrile 10%; 0.2–2.4 min, acetonitrile 10%–75%; 2.4–5.0 min, acetonitrile 75%–90%; 5.0–5.1 min, acetonitrile 90%–10%; and 5.1–6.5 min, 10% acetonitrile.

**Figure 2:**
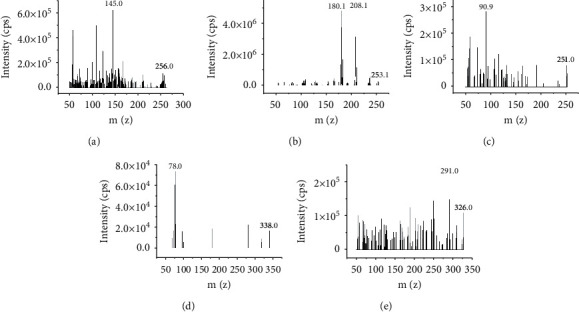
Mass spectrum of lamotrigine (a), oxcarbazepine (b), lacosamide (c), topiramate (d), and internal standard (e).

Nitrogen was adopted as the nebulizing gas (900 L/h), the capillary voltage was 2.9 kV, the ion source temperature was 150°C, and the nebulizing temperature was 550°C. Electrospray ionization (ESI) positive ion mode was used for lamotrigine, oxcarbazepine, lacosamide, and midazolam, and ESI-negative ion mode was used for topiramate. Multiple reaction monitoring (MRM) mode was performed for quantitative determination as follows: m/*z* 256⟶145 (cone voltage 44 V, collision voltage 36 V) for lamotrigine, m/*z* 253⟶180 (cone voltage 8 V, collision voltage 28 V) for oxcarbazepine, m/*z* 251⟶91 (cone voltage 4 V, collision voltage 20 V) for lacosamide, m/*z* 338⟶78 (cone voltage 40 V, collision voltage 24 V) for topiramate, and m/*z* 326⟶291 (cone voltage 25 V, collision voltage 25 V) for internal standard midazolam (Figure 2).

### 2.3. Control Solution Preparation

Lamotrigine, oxcarbazepine, lacosamide, topiramate, and midazolam (1.0 mg/mL) stock solutions were prepared with methanol-water (50 : 50, *v*/*v*). Stock solutions were diluted with methanol to prepare working solutions of different concentrations (50, 200, 1000, 5000, 10000, 20000, and 50000 ng/mL). All solutions were stored at 4°C and brought to room temperature before use.

### 2.4. Standard Curve Preparation

An appropriate amount of standard working solution of lamotrigine, oxcarbazepine, lacosamide, and topiramate was added to the rat plasma to prepare standard curves (5, 20, 100, 500, 1000, 2000, and 5000 ng/mL of the standard solution). The quality control (QC) samples were prepared in the same way as the plasma standard curve to obtain low, medium, and high concentrations (10, 900, and 4500 ng/mL) solutions.

### 2.5. Plasma Sample Pretreatment

The protein precipitation method was adopted for plasma sample pretreatment. Briefly, 50 *µ*L of rat plasma and 150 *µ*L of acetonitrile (containing midazolam 50 ng/mL) were added to a 1.5 mL Eppendorf tube, mixed, vortexed for 15 s (XW-80A Vortex Mixer, Kylin-Bell Lab Instruments, Haimen, China), and then centrifuged. After centrifugation at 13000 r/min for 10 min (Centrifuge 5804 R, Eppendorf AG, Hamburg, Germany), 150 *µ*L of the supernatant was transferred for glass vial detection [22, 23].

## 3. Method Verification

The verification method was established following the US Food and Drug Administration (FDA) Bioanalytical Method Verification Guidelines. The verification items included selectivity, matrix effect, linearity, precision, accuracy, recovery, and stability [24].

The method's selectivity was evaluated by analyzing blank rat plasma, blank plasma-spiked lamotrigine, oxcarbazepine, lacosamide, topiramate, and internal standard.

Calibration curves of lamotrigine, oxcarbazepine, lacosamide, and topiramate were constructed by analyzing spiked calibration samples on three separate days. The lower limit of quantification (LLOQ) was defined as the lowest concentration on the calibration curves, and the deviation should be within ±20%.

Blank rat plasma was extracted and spiked with the analyte at 10, 900, and 4500 ng/mL to evaluate the matrix effect. The corresponding peak areas were then compared to neat standard solutions at equivalent concentrations.

Accuracy and precision were assessed by determining QC in six replicates (10, 900, and 4500 ng/mL) over three days of validation testing. The precision is expressed as RSD.

The recovery of lamotrigine, oxcarbazepine, lacosamide, and topiramate was evaluated by comparing the peak area of extracted QC samples with those of reference QC solutions reconstituted in blank plasma extracts (*n* = 6).

The stability of lamotrigine, oxcarbazepine, lacosamide, and topiramate in rat plasma was evaluated by analyzing three plasma sample replicates at 10, 900, and 4500 ng/mL, exposed to different conditions. These results were compared with the freshly prepared plasma samples.

### 3.1. Pharmacokinetic Studies

Lamotrigine, oxcarbazepine, lacosamide, and topiramate were accurately weighed and dissolved in a dimethyl sulfoxide solvent to prepare 1 mg/mL solutions. The Animal Care Committee of Wenzhou Medical University approved all experimental procedures and protocols (Wydw 2019–0982). Eight rats were administered orally at a dose of 5 mg/kg. Blood samples (0.4 mL) were collected from the tail vein at 5, 15, 30 min and 1, 2, 4, 6, 8, 12, and 24 h postadministration. Samples were individually placed in a 1.5 mL Eppendorf tube and centrifuged at 5000 r/min for 10 min. Following centrifugation, a 150 *µ*L aliquot of plasma was removed and stored at −20°C. The pharmacokinetic parameters were obtained by DAS 2.0 software fitting.

## 4. Results

### 4.1. Selectivity

**Figure 3:**
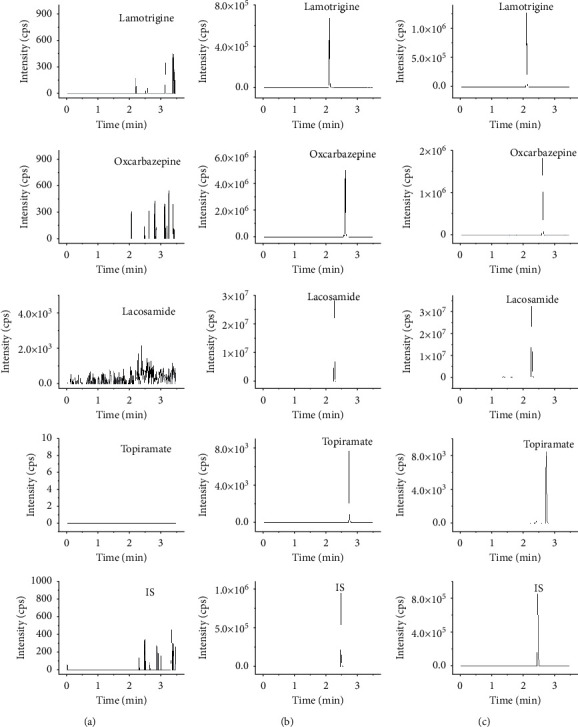
UPLC-MS/MS chromatograms of lamotrigine, oxcarbazepine, lacosamide, topiramate, and internal standard in rat plasma samples. Blank plasma sample (a); blank plasma spiked with lamotrigine and oxcarbazepine, lacosamide, topiramate, and internal standard (b); a rat plasma sample after the administration of the lamotrigine, oxcarbazepine, lacosamide, and topiramate (c).

Figure 3 shows the UPLC-MS/MS chromatograms of rat blank plasma samples and plasma samples spiked with lamotrigine, oxcarbazepine, lacosamide, and topiramate. The results showed that the endogenous substances in rat plasma samples had almost no effect on the determination of the four compounds.

### 4.2. Standard Curve Line

Lamotrigine, oxcarbazepine, lacosamide, and topiramate concentrations in rat plasma showed a linear relationship within the range of 5–5000 ng/mL. The standard curve equations are given in Table 1, where *y* represents the plasma concentration of lamotrigine, oxcarbazepine, lacosamide, and topiramate, and *x* represents the peak area of the drugs. The LLOQ of lamotrigine, oxcarbazepine, lacosamide, and topiramate in rat plasma was 5 ng/mL. The detection limit was 2 ng/mL with a signal-to-noise of 3.

### 4.3. Accuracy, Precision, Recovery, Matrix Effect

As given in Table 2, the intraday accuracy of lamotrigine, oxcarbazepine, lacosamide, and topiramate is within 92%–108%, and the interday accuracy is within 93%–109%; the RSDs of intraday and interday are less than 15%; the matrix effect is within 91%–105%, and the recovery is above 88%. It is concluded that the established UPLC-MS/MS method was suitable for pharmacokinetic studies of lamotrigine, oxcarbazepine, lacosamide, and topiramate.

### 4.4. Stability

The rat plasma stability tests were conducted at room temperature for 2 h, −20°C for 30 days, and three freeze-thaw cycles. The results in Table 3 show an accuracy within 92–108% and an RSD lower than 13%, indicating that lamotrigine, oxcarbazepine, lacosamide, and topiramate have good stability.

### 4.5. Pharmacokinetic Studies

**Figure 4:**
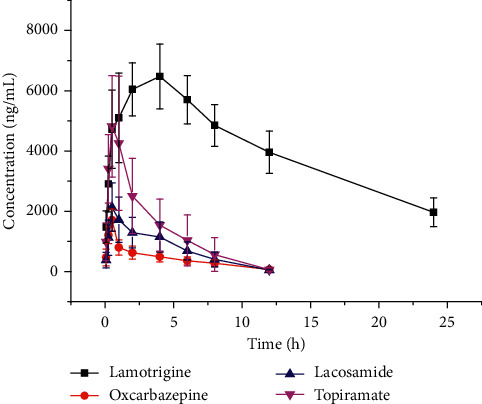
Plasma concentration-time curves of lamotrigine, oxcarbazepine, lacosamide, and topiramate in rats after oral administration.

The blood drug concentration over time curve is shown in Figure 4. The pharmacokinetic parameters peak drug concentration (*C*_max_), time to peak (*T*_max_), terminal elimination half-life (*t*_1/2_), area under the drug-time curve (AUC), clearance rate (CL), apparent volume of distribution (Vd), and mean residence time (MRT) were calculated according to the noncompartment model (Table 4). When the sample concentration was higher than 5000 ng/mL, blank rat plasma was diluted 10 times prior to processing.

## 5. Discussion

ESI-positive/negative electrode selection is often adopted in methodological studies. Lamotrigine, oxcarbazepine, and lacosamide are alkaloids more suitable for ESI-positive detection. However, topiramate is more suitable for ESI-negative electrode detection. Therefore, positive and negative ion switching modes were adopted to simultaneously detect lamotrigine, oxcarbazepine, lacosamide, and topiramate.

For LC conditions, the retention time of endogenous interferences should be as far away as possible from the examined compounds and internal standards [25, 26]. For this purpose, the relative chromatographic behavior of the column and mobile phase plays a decisive role; therefore, multiple columns and mobile phases were examined. First, BEH C18 and HSS T3 columns were tested. The HSS T3 (2.1 mm × 100 mm, 1.8 *μ*m) column had better separation and chromatographic peaks than BEH C18 and was chosen as the chromatographic column. This experiment also tried various mobile phases, including methanol-water, acetonitrile-water, methanol-0.1% formic acid, and acetonitrile-0.1% formic acid. Results showed that acetonitrile-0.1% formic acid had the best chromatographic peak shape.

Compared with the traditional HPLC method, UPLC-MS/MS was faster in the quantitative determination of lamotrigine, oxcarbazepine, lacosamide, and topiramate in plasma, with sample analysis completion in only 6.5 min. Lamotrigine, oxcarbazepine, lacosamide, and topiramate meet the requirements of the pharmacokinetic study due to their relatively low LLOQ (5 ng/mL). The *t*_1/2z_ of lamotrigine, oxcarbazepine, lacosamide, and topiramate after oral administration was 14.9 ± 11.4 h, 4.8 ± 2.0 h, 3.3 ± 2.8 h, and 2.5 ± 1.2 h, respectively. The CLz for lamotrigine, oxcarbazepine, lacosamide, and topiramate was 0.04 ± 0.01 L/h/kg, 0.9 ± 0.2 L/h/kg, 0.5 ± 0.2 L/h/kg, and 0.4 ± 0.2 L/h/kg, respectively. Therefore, rat' lamotrigine metabolism was slow compared to oxcarbazepine, lacosamide, and topiramate.

## 6. Conclusion

In this study, a UPLC-MS/MS method for the simultaneous determination of four antiepileptic drugs was established and applied to pharmacokinetics. The results showed that this method had good selectivity, high sensitivity, and an excellent linear relationship. Altogether, the presented work provides a reliable tool for monitoring antiepileptic drugs in plasma, which could be implemented in individual administration plan formulation, drug abuse monitoring, and pharmacokinetic studies.

## Figures and Tables

**Table 1:** The equations of the standard curve of lamotrigine, oxcarbazepine, lacosamide, and topiramate in rat plasma.

Compound	Equations	*R* ^2^	Range (ng/mL)	LLOQ (ng/mL)
Lamotrigine	*y* = 0.0005*x* + 0.0008	0.9987	5–5000	5
Oxcarbazepine	*y* = 0.0062*x* + 0.0055	0.9997	5–5000	5
Lacosamide	*y* = 0.0784*x* + 0.0691	0.9981	5–5000	5
Topiramate	*y* = 0.00004*x*−0.00003	0.9995	5–5000	5

**Table 2:** Accuracy, precision, matrix effect, and recovery of lamotrigine, oxcarbazepine, lacosamide, and topiramate in rat plasma.

Compound	Concentration (ng/mL)	Precision (RSD %)		Accuracy (%)		Matrix effect (%)	Recovery (%)
		Intraday	Interday	Intraday	Interday		
Lamotrigine	5	14.6	14.9	92.7	107.2	96.9	99.0
	10	8.3	12.1	101.7	101.2	95.6	94.0
	900	7.2	4.3	96.3	98.5	96.2	91.5
	4500	5.2	6.1	106.3	98.8	91.7	97.6
	5	11.0	13.1	107.6	93.4	103.2	96.0

Oxcarbazepine	10	6.1	10.7	99.4	105.8	99.9	93.8
	900	13.8	7.9	103.1	106.2	96.2	94.3
	4500	4.1	8.1	102.0	98.4	97.7	93.5
	5	9.1	8.8	93.6	108.2	101.8	92.2

Lacosamide	10	3.1	6.1	103.6	97.5	104.6	94.8
	900	5.6	3.4	97.7	104.2	98.3	98.4
	4500	4.8	5.8	99.1	102.9	98.3	96.7
	5	11.0	13.7	93.6	108.0	89.4	90.2

Topiramate	10	3.4	9.4	103.3	103.1	92.4	93.6
	900	9.1	5.5	97.7	98.6	95.8	88.8
	4500	3.0	8.0	95.3	96.1	99.0	90.4

**Table 3:** Stability of lamotrigine, oxcarbazepine, lacosamide, and topiramate in rat plasma under various storage conditions (*n* = 3).

Compound	Concentration (ng/mL)	Autosampler (4°C, 12 h)		Ambient (2 h)		–20°C (30 d)		Freeze-thaw	
		Accuracy	RSD	Accuracy	RSD	Accuracy	RSD	Accuracy	RSD
	10	97.2	4.7	107.9	5.1	96.7	10.1	106.7	8.9
Lamotrigine	900	105.1	4.8	100.0	5.4	93.1	11.6	95.3	10.4
	4500	99.9	3.0	95.2	2.7	101.3	2.8	107.2	3.8
	10	102.8	3.4	103.4	7.9	94.2	10.1	93.9	12.8
Oxcarbazepine	900	105.8	9.5	101.0	7.9	105.6	6.0	102.4	8.3
	4500	97.0	8.7	101.1	5.1	94.1	8.4	93.1	7.3
	10	102.2	3.2	99.2	3.1	94.9	9.1	97.1	10.5
Lacosamide	900	99.8	3.4	99.1	2.3	95.5	4.4	100.5	5.0
	4500	97.8	4.5	95.2	2.6	101.4	5.9	98.6	5.3
	10	96.4	7.5	96.3	9.2	92.9	9.6	97.2	11.7
Topiramate	900	98.0	8.9	103.5	5.5	104.0	7.0	102.5	8.4
	4500	105.1	4.9	95.2	9.8	94.8	3.4	90.4	10.2

**Table 4:** Pharmacokinetic parameters of lamotrigine, oxcarbazepine, lacosamide, and topiramate after oral administration in rats.

Parameters	Unit	Lamotrigine	Oxcarbazepine	Lacosamide	Topiramate
AUC_(0-*t*)_	ng/mL*∗*h	97824.5 ± 8269.9	4989.3 ± 1047.6	9270.3 ± 3231.7	16571.0 ± 7910.9
AUC_(0-∞)_	ng/mL*∗*h	154786.8 ± 86468.6	6364.0 ± 2161.0	10586.9 ± 4015.8	17329.1 ± 8451.5
MRT_(0-*t*)_	h	9.4 ± 0.7	3.6 ± 0.3	3.6 ± 0.5	3.0 ± 0.6
MRT_(0-∞)_	h	19.8 ± 8.4	5.1 ± 1.9	3.6 ± 0.4	3.1 ± 0.6
*t*_1/2z_	h	14.9 ± 11.4	4.8 ± 2.0	3.3 ± 2.8	2.5 ± 1.2
*T*_max_	h	2.8 ± 1.5	0.5 ± 0.1	0.8 ± 0.5	0.6 ± 0.3
CLz	L/h/kg	0.04 ± 0.01	0.9 ± 0.2	0.5 ± 0.2	0.4 ± 0.2
Vz	L/kg	0.7 ± 0.1	5.7 ± 2.9	2.2 ± 1.3	1.5 ± 1.7
*C*_max_	ng/mL	6776.1 ± 916.2	1713.1 ± 361.3	2258.2 ± 686.6	5203.4 ± 1650.8

## Data Availability

The data used to support the findings of this study are included within the article.
